# Carotenoids in Skin Photoaging: Unveiling Protective Effects, Molecular Insights, and Safety and Bioavailability Frontiers

**DOI:** 10.3390/antiox14050577

**Published:** 2025-05-11

**Authors:** Yingchao Ma, Chengxiang Li, Wanping Su, Zhongshi Sun, Shuo Gao, Wei Xie, Bo Zhang, Liying Sui

**Affiliations:** 1 Key Laboratory of Marine Resource Chemistry and Food Technology (TUST), Ministry of Education, Tianjin 300457, China; yingchaoma@tust.edu.cn (Y.M.); xiewei@tust.edu.cn (W.X.); 2 Asian Regional Artemia Reference Center, College of Marine and Environmental Sciences, Tianjin University of Science and Technology, Tianjin 300457, China; licx@mail.tust.edu.cn (C.L.); suwanping1005@126.com (W.S.); sun_zhongshi@yeah.net (Z.S.); gaoshuo20020102@163.com (S.G.); 3 Tsinghua University Institute of TCM-X, Beijing 100084, China; zhangbo@tjab.org

**Keywords:** carotenoids, photoaging, oxidative stress, ultraviolet radiation, bacterioruberin

## Abstract

Skin photoaging, driven primarily by ultraviolet radiation, remains a critical dermatological concern. Carotenoids, a class of natural pigments with potent antioxidant properties, have emerged as promising agents for preventing and mitigating photoaging. This review comprehensively integrates current understanding regarding the triggers of skin photoaging, oxidative stress and their associated signal pathways, the photoprotective roles and mechanisms of carotenoids, as well as their bioavailability. Common C_40_ carotenoids, such as β-carotene, lycopene, astaxanthin, lutein, and zeaxanthin demonstrate remarkable antioxidant activity, primarily attributed to their conjugated double bond structures. Many studies have demonstrated that both oral and topical administration of these C_40_ carotenoids can effectively alleviate skin photoaging. Specifically, they play a crucial role in promoting the formation of a new skin barrier and enhancing the production of collagen and elastin, key structural proteins essential for maintaining skin integrity and elasticity. Mechanistically, these carotenoids combat photoaging by effectively scavenging reactive oxygen species and modulating oxidative stress responsive signal pathways, including MAPK, Nrf2, and NF-κB. Notably, we also anticipate the anti-photoaging potential of novel carotenoids, with a particular emphasis on bacterioruberin, a C_50_ carotenoid derived from halophilic archaea. Bacterioruberin exhibits a superior radical scavenging capacity, outperforming the conventional C_40_ carotenoids. Furthermore, when considering the application of carotenoids, aspects such as safe dosage, bioavailability, and possible long term usage issues, including allergies and pigmentation disorders, must be taken into account. This review underscores the anti-photoaging mechanism of carotenoids, providing strategies and theoretical basis for the prevention and treatment of photoaging.

## 1. Introduction

The skin, composed of the epidermis, dermis, and subcutaneous tissues, plays a crucial role in barrier function, temperature regulation, tactile perception, and immune monitoring [1]. Skin aging is the most visible manifestation of the aging process, and extrinsic aging is a significant subtype within it [2]. Extrinsic aging is primarily induced by long-term exposure to various environmental factors, such as ultraviolet (UV) radiation, air pollution, ionizing radiation, and toxins. This exposure leads to a series of skin problems, including sunburn, roughness, sagging, dullness, deep wrinkles, telangiectasia, mottled pigmentation, reduction in elasticity, delayed wound healing, and an increased susceptibility to cancer [3,4]. Among these factors, UV radiation is responsible for 80–90% of the visible signs of extrinsic aging, making it one of the most common causes of photoaging [5,6]. When the skin is exposed to UV rays, it stimulates cells to generate excessive reactive oxygen species (ROS), which disrupt the normal redox balance and trigger an oxidative stress response. This oxidative stress can cause damage to cellular components, including DNA, proteins, and lipids, ultimately accelerating the photoaging process.

**Figure 1:**
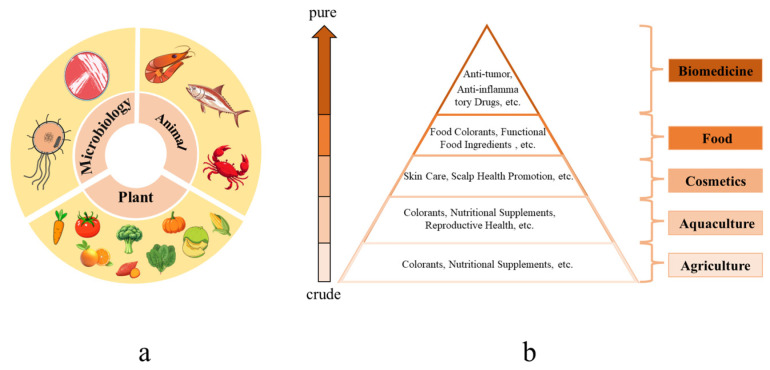
Sources and applications of carotenoids. (**a**) Source of carotenoids. (**b**) Applications of carotenoids.

In the quest to combat skin photoaging, natural antioxidants have emerged as promising candidates. Compounds such as luteolin [7], tannic acid [8], and protocatechuic acid [9], chlorogenic acid and resveratrol [10], curcumin [11], and catechins [12] have been shown to effectively scavenge ROS, reduce oxidative damage, and delay the progression of photoaging. Among the natural antioxidants, carotenoids, widely distributed in microorganisms (e.g., microalgae, halophilic bacterial, and archaea) [13], plants (e.g., carrot, tomato, broccoli, corn, pumpkin, lettuce, orange, cherry, mango) [14,15,16], and aquatic animals (e.g., fish, shrimps, and crabs) [17] have gained significant interest due to their diverse biological activities. They possess multiple functions, including anti-inflammation [18], antitumor [19,20,21], anti-diabetes [22], and blood vessels protection [23], with their antioxidant activity being particularly remarkable [24] (Figure 1). A substantial number of studies have evidenced that carotenoids can alleviate photoaging [25,26,27]. However, there are no comprehensive reviews on the existing knowledge regarding the effects of carotenoids on photoaging. This review aims to comprehensively explore the effect of carotenoids on photoaging and provide valuable insights into scientific research, clinical practice, and public health improvement.

## 2. Skin Photoaging

### 2.1. Ultraviolet Radiation as the Primary Trigger of Skin Photoaging

**Figure 2:**
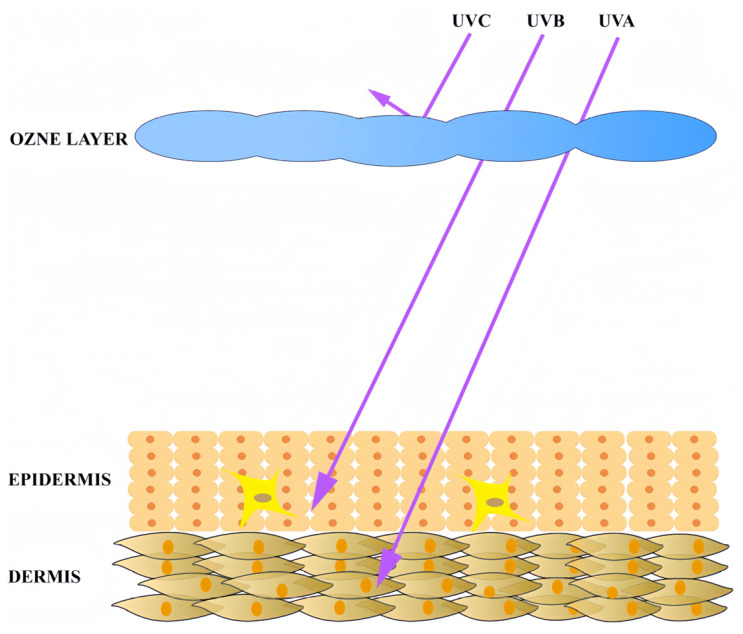
Ultraviolet radiation penetration in skin tissue.

UV radiation, the principal contributor to skin aging, is categorized into three subtypes based on wavelength: UVA (315–400 nm), UVB (280–315 nm), and UVC (100–280 nm) [28]. The ozone layer absorbs nearly all UVC and over 95% of UVB, allowing only approximately 5% of UVB and 95% of UVA to reach the Earth’s surface [29,30]. UVB primarily affects the epidermal layer of the skin. Approximately 70% of UVB is absorbed by the stratum corneum, 20% reaches differentiated epidermal regions (e.g., the granular layer), and only 10% penetrates to the superficial dermis [31] (Figure 2). UVB directly induces DNA damage through the formation of cyclobutane pyrimidine dimers (CPDs) and 6-4 pyrimidine photoproducts (6-4PPs), leading to genomic instability and cell death [32].

UVA, with its longer wavelength, exhibits deeper skin penetration and plays a crucial role in photoaging by targeting both the epidermis and dermis. In the epidermis, UVA induces oxidative stress, DNA damage, and apoptosis in keratinocytes, the most abundant cells in the epidermis [33]. This process contributes to impaired skin barrier function and an increased risk of carcinogenesis, such as melanoma [34]. Within the dermis, collagen and elastin, the key structural proteins responsible for skin firmness and elasticity, are degraded upon UVA exposure [35,36]. Studies demonstrate that UVA disrupts collagen fiber synthesis and promotes elastic fiber fragmentation [37,38,39]. Furthermore, UVA stimulates dermal cells to generate excessive ROS, triggering oxidative stress, inflammatory cascades, and apoptotic pathways. These cumulative effects underscore UVA as the critical driver of skin photoaging.

### 2.2. Skin Photoaging and Oxidative Stress

Continuous exposure to UV radiation induces excessive accumulation of ROS in cutaneous tissues. ROS are a group of atoms or molecules possessing one or more unpaired electrons and can be classified into radical oxides (e.g., superoxide anion radical and hydroxyl radical) and non-radical oxides (e.g., singlet oxygen and hydrogen peroxide) [40]. Radical oxides have the ability to break single-strand DNA, whereas non-radical oxides typically oxidize DNA bases [41,42]. Given that excess ROS cannot be scavenged in a short period of time, a chemical imbalance between the production and consumption of oxidants in biological systems is induced, which activates the oxidative stress response [43]. This activation results in the oxidation of bio-macromolecules and the activation of signal pathways, ultimately leading to a series of skin photoaging symptoms [44,45,46]. 

#### 2.2.1. Bio-Macromolecule Damage by Oxidative Stress

DNA, lipids, and proteins are crucial biological macromolecules in organisms, involving a wide array of biochemical reactions. UV radiation generates ROS, which can induce oxidative damage on nuclear DNA. This damage is induced through diverse molecular pathways, including single-base modifications (notably purine alterations), DNA interstrand cross-links, DNA–protein cross-links, and the generation of apurinic/apyrimidinic sites via depurination or depyrimidination processes. As a consequence, large quantities of thymine glycol and 8-hydroxyguanine are produced [45,47]. Base excision repair is a mechanism capable of rectifying this type of DNA damage. However, with advancing age, the efficiency of various DNA repair mechanisms declines, including nucleotide excision repair, base excision repair, double-strand break repair, and mismatch repair [48]. The decrease in age-related repair efficiency exacerbates the accumulation of DNA damage induced by ROS.

In addition to DNA damage, ROS also target cell membrane lipids. Unsaturated fatty acids with double bonds in the cell membrane are particularly susceptible to attack by hydroxyl radicals (·OH) and peroxynitrite (ONOO^−^). For instance, the hydroxyl radical (·OH) initiates lipid peroxidation cascades through hydrogen atom abstraction from polyunsaturated fatty acid, generating lipid radicals. Under aerobic conditions, these lipid radicals initiate lipid peroxidation. Lipid peroxidation has been shown to alter the membrane structure, affect its fluidity, and disrupt its integrity [49]. The lipid peroxides formed during this process can further break down into polyreactive aldehydes. These aldehydes react with DNA bases, generating unrepairable polymers that may ultimately lead to the development of diseases. Malondialdehyde (MDA), a common product of lipid peroxidation, plays a significant role in cellular damage. It causes cross-linking polymerization between membrane proteins and phospholipids, directly disrupting the phospholipid bilayer of the cell membrane [50]. This disruption leads to irreversible denaturation of membrane proteins. Moreover, MDA can react with DNA bases, potentially causing gene mutations [51].

ROS also have a detrimental impact on proteins. Hydrogen peroxide (H_2_O_2_) and superoxide anions (O_2_^−^) can directly interact with the active center of cysteine containing proteins, resulting in protein inactivation [52]. Additionally, ROS can damage proteins through various means. They can directly attack the protein backbone, oxidize amino acid residues, cleave peptide bonds, form protein aggregates, and generate oxidative stress by-products [53,54]. These effects lead to the loss of activity of many enzymes and the disruption of metabolic pathways.

In summary, UV-induced ROS can cause extensive oxidative damage to DNA, lipids, and proteins. The senescence-associated attenuation of DNA repair systems further exacerbates oxidative damage. The damage to lipids and proteins not only disrupts cellular structure and function but also has the potential to cause genetic mutations, highlighting the complex effects of ROS in the process of skin photoaging.

#### 2.2.2. Signal Pathways Activated by Oxidative Stress

Studies have shown that ROS, acting as second messengers, can activate diverse signal transduction pathways. These pathways further regulate the expression of downstream genes, ultimately resulting in skin photoaging.

*Activation of the MAPK Signal Pathway*. UV irradiation generates excessive ROS, which initiate photo damage through sequential activation of the mitogen-activated protein kinase (MAPK) signaling cascade [55]. This process begins with ROS-mediated phosphorylation of the epidermal growth factor receptor (EGFR), triggering downstream MAPK activation. The MAPK family, comprising serine/threonine kinases, is subdivided into three major subfamilies based on stimulus specificity and functional divergence, including extracellular signal-regulated kinases (ERK), p38 MAPK, and c- Jun N-terminal kinases (JNK) [56]. Among these, UVA-induced ROS activate p38 and JNK through phosphorylation cascades originating from cytokine receptors, EGFR, and Ras GTPase [57]. These kinases phosphorylate upstream regulators (e.g., ASK1, MKK4/7), culminating in nuclear translocation of activated MAPKs. Activated MAPK proteins enter the nucleus and activate multiple transcription factors, such as adaptor protein complex-1 (AP-1), nuclear factor kappa-B (NF-κB), cyclooxygenase-2 (COX-2), and myc proto-oncogene protein (c-Myc) [57,58,59]. This activation induces the expression of matrix metalloproteinases (MMPs), upregulates the expression of inflammatory factors like interleukin-1 beta (IL-1β), IL-6, and tumor necrosis factor alpha (TNF-α), and downregulates the expression of transforming growth factor beta (TGF-β), resulting in photo damage [60]. Critically, these transcriptional events establish a self-reinforcing loop, ROS-induced MAPK activation enhances inflammatory mediator production, which further elevates intracellular ROS via NADPH oxidase activation [61].

*Activation of the Nrf2 Signal Pathway*. The nuclear factor erythroid 2-related factor 2 (Nrf2) signal pathway constitutes a master regulatory system for cellular redox homeostasis. Under physiological conditions, Nrf2 is constitutively sequestered in the cytoplasm through its interaction with Kelch-like ECH-associated protein 1 (Keap1), which functions as a substrate adaptor for the Cullin 3 (Cul3)-based E3 ubiquitin ligase complex [62,63]. This Keap1-Cul3-E3 ligase complex directs Nrf2 for polyubiquitination and subsequent proteasomal degradation, thereby maintaining low basal levels of Nrf2 under unstressed conditions. Mild oxidative stress triggers a biphasic regulatory mechanism: low-dose ROS promote the dissociation of the Keap1-Nrf2 complex, facilitating their nuclear co-translocation. Within the nucleus, Nrf2 heterodimerizes with small Maf proteins (e.g., MafG/K) and binds to antioxidant response elements (AREs) in the promoter regions of target genes, initiating the transcription of phase II detoxifying enzymes (e.g., NQO1, HO-1) and antioxidant proteins (e.g., glutathione synthetase), which collectively restore redox equilibrium [64,65,66]. Paradoxically, sustained oxidative stress disrupts this adaptive response through redox-sensitive modifications. Excessive ROS induce hyperoxidation of critical cysteine residues (e.g., Cys151, Cys273, Cys288) within Keap1, stabilizing its interaction with Nrf2 and enhancing Cul3-mediated ubiquitination [67]. Concurrently, oxidative modifications of Nrf2 itself (e.g., cysteine sulfenylation) impair its nuclear translocation and DNA-binding capacity, leading to transcriptional silencing of antioxidant genes. This dual inhibition mechanism exacerbates photoaging by depleting cellular defenses against UV-induced oxidative damage.

*Activation of the NF-κB Signal Pathway*. The NF-κB pathway serves as a pivotal mediator in cellular responses to oxidative stress, exhibiting dual regulatory roles in both pro-inflammatory and antioxidant processes [68,69]. As a transcription factor, NF-κB governs critical biological functions including cell proliferation, apoptosis, and stress adaptation to diverse pathological stimuli [70,71]. Under basal conditions, NF-κB remains sequestered in the cytoplasm as an inactive complex through its interaction with inhibitor proteins IκB, which binds specifically to the Rel homology domain (RHD) of NF-κB [72]. Upon oxidative stress, excessive ROS activate IκB kinase (IKK), triggering phosphorylation of serine residues on IκB. The post-translational modification, typically phosphorylation at specific serine residues on IκB, serves as a molecular signal that targets IκB for proteasomal degradation. As a result, the NF-κB heterodimer, composed of p50 and p65 subunits, is liberated from its inhibitory binding to IκB. Once free, the NF-κB heterodimer exposes its nuclear localization signals. This exposure enables the NF-κB complex to interact with importin proteins, facilitating its translocation into the nucleus, where it can then regulate gene expression. Within the nucleus, NF-κB initiates transcription of pro-inflammatory cytokines (e.g., IL-2, IL-8) and enzymes such as cyclooxygenase-2 (COX-2), thereby driving inflammation and tumorigenesis [72]. Notably, this inflammatory cascade establishes a feedforward loop, as secreted cytokines (e.g., TNF-α) further amplify NF-κB activation through receptor-mediated signaling. Paradoxically, NF-κB activation also confers adaptive antioxidant responses under oxidative stress. It transcriptionally upregulates antioxidant enzymes including superoxide dismutase (SOD) and glutathione peroxidase (GPx), whose enzymatic activities counteract ROS accumulation and restore redox homeostasis. The balanced NF-κB activity determines cellular fate under oxidative stress conditions [73,74].

## 3. The Anti-Photoaging Effect and Mechanism of Carotenoids

Oxidative stress typically activates the skin’s antioxidative system, which includes antioxidases and non-enzymatic antioxidants. Research has indicated that singlet oxygen (^1^O_2_) generated by UV radiation can deactivate antioxidases [75,76]. In such circumstances, non-enzymatic antioxidants, like carotenoids, glutathione, and coenzyme Q10, play a crucial role in responding to oxidative stress. Among these, carotenoids exhibit distinct advantages over other antioxidants.

**Figure 3:**
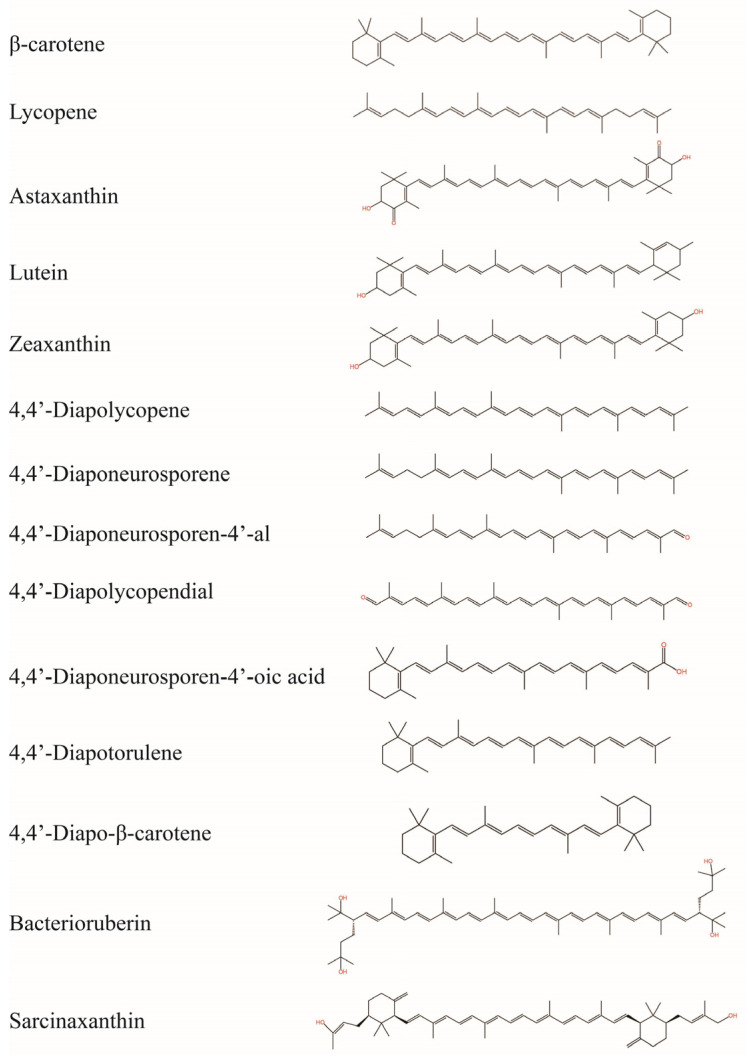
Chemical structures of related carotenoids.

Carotenoids, as natural products, are widely distributed in various living organisms, including plants, microalgae, bacteria, fungi, and archaea. To date, more than 800 natural carotenoids have been identified [77]. This wide distribution makes them easily accessible. Structurally, carotenoids are composed of isoprene units, a structure that confers good stability (Figure 3). This structure also endows carotenoids with a certain degree of lipophilicity, enabling them to better bind to cell membranes and improve their bioavailability. In addition, carotenoid molecules feature a large number of conjugated double bonds. For example, β-carotene and lycopene have eleven conjugated double bonds, while astaxanthin and bacterioruberin have thirteen [78]. The antioxidant efficacy of carotenoids is fundamentally governed by the number of conjugated double bond, wherein a higher density proportionally amplifies their antioxidant capacity. This is because the conjugated double bond structure allows for efficient electron delocalization, which is crucial for neutralizing reactive species [79]. Carotenoids has been confirmed to possess a potent radical scavenging capacity and can effectively eliminate a wide spectrum of radicals, including 1,1-Diphenyl-2-picrylhydrazyl (DPPH), 2,2′-azino-bis (3-ethylbenzothiazoline-6-sulfonic acid) (ABTS), and ROS. Many studies have confirmed that carotenoids have a greater ability to scavenge DPPH, ABTS, and ROS radicals compared to the chemical antioxidants such as ascorbic acid, α-tocopherol, and butylhydroxytoluene (BHT) [80,81,82,83]. According to their elemental composition, carotenoids can be classified into two main categories: carotenes, which consist solely of hydrocarbon atoms, and xanthophyll, which contain hydrocarbon and oxygen atoms. Additionally, the terminal groups of carotenoids, such as hydroxyl and carbonyl groups, can further enhance their antioxidant capacity. For instance, astaxanthin has over 10 times the ^1^O_2_ quenching capacity of β-carotene [84]. Except for the antioxidant activity, carotenoids also have antitumor [19,85,86,87,88], antibacterial [89,90,91], antihemolytic [86], photoprotective activity [24,92], immunomodulation [93], regulation of lipid metabolism [94], anti-atherosclerosis [95], vision protection [95], etc., allowing its wide application in food, dietary supplement, and pharmaceutical industries. Moreover, studies have shown that appropriate intake or topical application of carotenoids can effectively protect the skin from UV radiation, showing great promise in anti-photoaging [96].

### 3.1. The Anti-Photoaging Effect and Mechanism of C_40_ Carotenoids

C_40_ carotenoids are highly prevalent, with β-carotene, lycopene, astaxanthin, lutein, and zeaxanthin being prominent examples. These carotenoids are extensively utilized in various industrial applications, thus attracting numerous research efforts. Among them, β-carotene and lycopene fall into the category of carotene, while astaxanthin, lutein, and zeaxanthin are classified as a xanthophyll. C_40_ carotenoids such as lutein, zeaxanthin, and lycopene are excellent antioxidants, a fact that has also been demonstrated by testing the oxidized products of these carotenoids in the blood [97]. Studies have indicated that C_40_ carotenoids play a significant role in alleviating cardiovascular diseases, neurodegenerative disorders, obesity, and diabetes. In addition, carotenoids (e.g., β-carotene, lycopene, and lutein) and their oxidation products have been found in human skin [98], and they have been reported to possess anti-photoaging properties (Table 1).

#### 3.1.1. The Anti-Photoaging Effects and Mechanism of β-Carotene

β-Carotene (C_40_H_56_, molecular weight of 536.87), a prominent member of the carotenoid family, is ubiquitously distributed in nature. It has been mainly found in vegetables (e.g., carrots, potatoes, spinach, lettuce, broccoli) and fruits (e.g., orange, cantaloupe, mango) [14]. As a pro-vitamin A carotenoid, β-carotene also serves as a critical dietary precursor for retinol biosynthesis, undergoing enzymatic cleavage to meet the body’s vitamin A requirements [99]. The antioxidant properties of β-carotene are intrinsically linked to its unique polyene structure, featuring 11 conjugated double bonds and two β-ionone rings [100]. In addition to its antioxidant properties, β-carotene demonstrates diverse biological functions, such as anti-inflammatory effects [101], angiogenesis [102], photoprotective activity [103], and immunomodulation [104], enabling its extensive utilization in cosmetics, pharmaceuticals, and nutraceutical industries.

**Figure 4:**
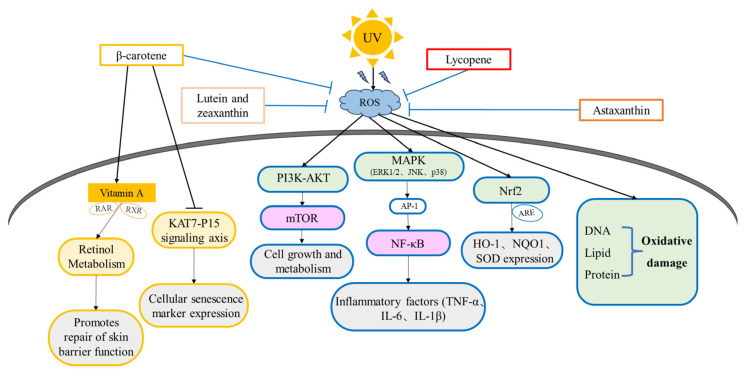
The mechanisms of C_40_ carotenoids on anti-photoaging. Note: Blue boxes indicate signal pathways common to the three carotenoids; orange boxes indicate β-carotenoid-specific signal pathways. UV: ultraviolet; ROS: reactive oxygen species; RAR: retinoic acid receptor; RXR: retinoid X receptor; mTOR: mammalian target of rapamycin; ERK1/2: extracellular signal-regulated kinase 1/2; JNK: c-Jun N-terminal kinase; p38: p38 mitogen-activated protein kinase; AP-1: activator protein 1; Nrf2: nuclear factor erythroid 2-related factor 2; ARE: antioxidant response element; HO-1: heme oxygenase 1; NQO1: NAD (P) H: quinone oxidoreductase 1; SOD: superoxide dismutase; NF-κB: nuclear factor kappa-light-chain-enhancer of activated B cells; TNF-α: tumor necrosis factor alpha; IL-6: interleukin 6; IL-1β: interleukin 1 beta.

Clinical evidence highlights β-carotene’s efficacy as a systemic photoprotective agent (Table 1). Research displayed that β-carotene can improve facial wrinkles and elasticity, reduce erythema, increase mRNA levels of collagen I, inhibit the expression of MMP-9, and reduce UV-induced DNA damage, thereby delaying skin aging [105,106,107]. Moreover, in patients with erythropoietic protoporphyria (EPP) and polymorphic light eruption (PMLE), prolonged supplementation (>10 weeks) of β-carotene at doses exceeding 12 mg/day significantly reduces UV-induced erythema and photo-dermatosis severity [108,109]. The delayed progression of this therapeutic effect is associated with the accumulation of β-carotene in tissues. To achieve optimal skin photoprotection, sustained treatment is necessary [110]. Mechanistic studies further clarify the multimodal actions of β-carotene. Firstly, β-carotene can scavenge ^1^O_2_ and inhibit lipid peroxidation. Additionally, in vitro models demonstrate that β-carotene supplementation enhances cellular antioxidant capacity by activating catalase (CAT) and SOD while concurrently downregulating MMP expression in dermal fibroblasts [111,112]. These regulations preserve extracellular matrix (ECM) architecture by reducing collagen degradation and counteracting UV-induced photoaging. In terms of mechanism, β-carotene can delay cellular aging by activating the Nrf2/ARE signal pathway, inducing the expression of antioxidant enzymes and phase II detoxification enzymes, and enhancing cellular antioxidant and detoxification capacity [113]. In addition, research showed that β-carotene can regulate the process of autophagy and apoptosis through the PI3K/AKT/mTOR signal pathway and reduce cell damage [114]. Furthermore, Wu et al. found that β-carotene can reduce malondialdehyde, TNF-α, and IL-6 levels and increase glutathione peroxidase and superoxide dismutase levels through NF-κB, MAPK, and Nrf 2 signal pathways [115]. β-carotene can also be converted to vitamin A through the KAT7-P15 signaling axis, which can be involved in the metabolism of retinol in order to slow down the aging of cells [116,117,118] (Figure 4). Current interventional trials support β-carotene’s role in mitigating cutaneous damage in these contexts, though precise dosing protocols require further standardization [119].

#### 3.1.2. The Anti-Photoaging Effects and Mechanism of Lycopene

Lycopene (C_40_H_56_, molecular weight of 536.87), a non-oxygenated carotenoid, exists as needle-like deep red crystals in its pure form. This compound is predominantly found in red fruits and vegetables such as tomatoes, watermelon, apricots, and guava, with tomatoes exhibiting the highest natural abundance [120,121]. Recent advances in metabolic engineering and synthetic biology have enabled the heterologous biosynthesis of lycopene in engineered microbial systems, including *Escherichia coli* [122], *Saccharomyces cerevisiae* [123], *Yarrowia lipolytica* [124], and *Deinococcus radiodurans* [125]. Through targeted modification of microbial metabolic pathways, these microorganisms have been transformed into efficient cellular factories for high-yield lycopene production.

Although lycopene shares the same molecular formula as β-carotene, their structures exhibit distinct differences. Specifically, lycopene features a linear structure, whereas β-carotene has two rings at its terminals. Lycopene, with its structural configuration of eleven conjugated and two non-conjugated double bonds, endows it with exceptional radical scavenging capacity. Among dietary carotenoids, lycopene demonstrates superior ^1^O_2_ quenching efficiency, surpassing β-carotene and vitamin E by 47-fold and 100-fold, respectively [126,127]. Lycopene has an important role in slowing down apoptosis and skin aging (Table 1). It reduces ROS, β-galactosidase and advanced glycation end products (AGE), and increases mitochondrial membrane potential and attenuates apoptosis [128,129]. It improves skin elasticity, firmness, brightness, tone as well as fine lines and wrinkles, reduces erythema, decreases inflammatory oxidative indicators, enhances anti-elastase activity, anti-melanogenic activity, and anti-tyrosinase activity, inhibits melanin precursor darkening, and reduces UV-induced skin damage [130,131,132,133,134,135,136]. Moreover, lycopene combined with vitamin E enhances the inhibition of MMP-1 expression after UVA irradiation [137]. Mechanistically, lycopene attenuates oxidative stress and apoptosis by activating the PI3K/Akt/Nrf2 signal pathway [138], upregulating the expression of antioxidant and anti-apoptotic proteins and downregulating the expression of pro-apoptotic proteins [139]. In addition, lycopene protects the skin by inhibiting the MAPK and NF-κB signal pathways and reducing the inflammatory response [140] (Figure 4). These findings collectively establish lycopene-rich dietary interventions as effective strategies against acute photo damage and potential long-term photoaging sequelae.

#### 3.1.3. The Anti-Photoaging Effects and Mechanism of Astaxanthin

Astaxanthin (C_40_H_52_O_4_, molecular weight of 596.85), a xanthophyll-class carotenoid, is a lipid-soluble red pigment. Ubiquitously distributed in nature, astaxanthin is biosynthesized by microalgae (e.g., *Haematococcus pluvialis*) and bacteria, and it accumulates in higher trophic organisms such as crustaceans through dietary transfer [141,142]. Commercial production primarily relies on microbial fermentation of *H. pluvialis* [143], rather than direct extraction from other organisms, due to yield and purity advantages. Astaxanthin is characterized by a unique molecular architecture: two β-ionone rings at the termini interconnected by a conjugated polyene chain of four isoprene units. This structural configuration, particularly the extended conjugated double-bond system and terminal cyclic moieties, confers exceptional antioxidant capacity. Pharmacologically, astaxanthin exhibits multiple bioactivities, including antitumor [144,145], antidiabetic [146], anti-atherosclerotic [147], and anti-inflammatory effects [148].

Notably, its protective role against UV-induced photoaging has garnered significant attention (Table 1). In vivo studies using UVA-irradiated hairless mice demonstrated that oral astaxanthin administration (10–40 mg/kg/day) reduced wrinkle formation by 30–50%, suppressed ROS generation and enhanced dermal collagen density via MMP-13 downregulation and aquaporin-3/steroid sulfatase modulation [77,78]. Astaxanthin is also able to improve skin elasticity and hydration, as well as improve wrinkles, elasticity, transepidermal water loss (TEWL), moisture content, and sebum oil content [149,150]. In vitro analyses further revealed the effects of astaxanthin, which was found to inhibit cellular damage caused by free radicals, reduce UVA radiation-induced increases in IL6 expression levels, and reduce ROS generation [151]. Inhibition of keratinocyte apoptosis occurred through the reduction in iNOS and COX-2 [152]. Mechanistically, astaxanthin can act through PI3K/Akt, Nrf2, NF-κB, and MAPK signal pathways. For example, it has been found that astaxanthin reduces ROS production and inhibits apoptosis in mouse photoreceptor cells through the PI3K/Akt/Nrf2 signal pathway [153]. Hama et al. found that astaxanthin reduced UV-induced collagen degradation and elastic fiber damage through Nrf2, NF-κB, and MAPK signal pathways, reduced the expression of MMPs (such as MMP-1, MMP-3, MMP-9), and resulted in delaying skin aging [154] (Figure 4). Current evidence substantiates astaxanthin as a potent nutraceutical agent for integrative dermatological interventions.

#### 3.1.4. The Anti-Photoaging Effects and Mechanism of Lutein and Zeaxanthin

Lutein and zeaxanthin (C_40_H_56_O_2_, molecular weight of 566.88) are two important carotenoids, primarily of plant origin, that have a variety of health benefits, especially in eye health [155]. Despite sharing an identical molecular formula, these isomers are distinguished by a single positional difference in the conjugated double-bond system: lutein contains one β-ionone ring and one ε-ionone ring, whereas zeaxanthin possesses two β-ionone rings. In rat tracheal epithelial cells, their synergetic scavenging of ROS enhances cellular viability, suppresses NF-κB mediated inflammatory signaling (including IL-1β and COX-2 downregulation), and mitigates UVA-induced DNA damage [156,157] (Figure 4). In skin aging models, these carotenoids exhibit photoprotective efficacy by improving epidermal barrier function (enhanced hydration and elasticity) and reducing wrinkle formation. Notably, murine studies demonstrate their capacity to inhibit UVB-triggered epidermal hyperplasia and acute inflammatory responses, while alleviating xerosis [158,159,160] (Table 1).

**Table 1:** The anti-photoaging effect and mechanism of C_40_ carotenoids.

Name	Antioxidant Capacity	In Vitro Experiment	In Vivo Experiment	Signal Pathways	References
β-carotene	Singlet oxygen (all-E-isomer-rich, IC_50_ 0.38 μg/mL; Z-isomer-rich IC_50_ = 0.95 μg/mL)	Human skin fibroblasts (HSFs), human229 neonatal skin fibroblasts (NB1RGB), and B16274 mouse melanoma cells: enhancing hyaluronic acid production, promoting proliferation, anti-elastase activity, anti-melanogenic activity and anti-tyrosinase activity, inhibition of type I collagen production, and inhibition of melanin precursor darkening	-	-	[130]
	-	Keratinocyte: inhibition of UVA-induced ECM degradation and enhancement of UVA-induced expression of tanning-related protease-activated receptor 2, promotes cell differentiation	-	-	[117,118]
	-	Mesenchymal stem cells (MSCs): reducing the expression of cellular senescence markers (e.g., SA-β-gal, p21, p53), enhancing cellular antioxidant capacity, and reducing oxidative stress-induced cell damage	C57 mice: improving the aging state of many tissues and organs, reducing expression of inflammatory factors	KAT7-P15	[116]
	-	Human mammary cancer cells (MCF-7) and human hepatocellular carcinoma cells (HepG 2): activating ARE, inducing the expression of antioxidant enzymes and phase II detoxification enzymes, and enhancing cellular antioxidant and detoxification capacity	-	Nrf2/ARE	[113]
	-	Rat Small Intestine Crypt Epithelial Cells (IEC): Down-regulation of caspase-3, Bax levels and LC3II/I ratio, and up-regulation of Bcl-2 and p62 levels were used to reduce autophagy and inhibit apoptosis	-	PI3K/AKT/mTOR	[114]
	-	-	Mice: decreasing malondialdehyde, TNF-α and IL-6 levels, and increasing glutathione peroxidase and superoxide dismutase levels	NF-κB/MAPK/Nrf2	[115]
	-	-	Healthy female subjects: improving facial wrinkles and elasticity, increases collagen type I mRNA levels, reduces UV-induced DNA damage	-	[105]
	-	-	Hairless mice: inhibiting MMP-9 expression and reducing skin wrinkles and sagging	-	[107]
	-	-	11 male and 11 female subjects: protecting human skin from UVA and UVB-induced erythema, reducing serum lipid peroxidation	-	[106]
Lycopene	Singlet oxygen (all-E-isomer-rich IC_50_ 0.26 μg/mL, Z-isomer-rich IC_50_ 1.06 μg/mL)	HSF: Enhancing hyaluronic acid production, promoting proliferation	Anti-elastase activity, anti-melanogenic activity, and anti-tyrosinase activity, inhibition of melanin precursor darkening	-	[130]
	-	-	Human oral intake: reducing erythematous reaction	-	[131]
	-	Chinese hamster ovary cell (M146L cell): reduction in oxidative stress and apoptosis, upregulation of antioxidant and anti-apoptotic proteins, downregulation of pro-apoptotic proteins	-	PI3K/Akt/Nrf2	[139]
	-	Macrophages: inhibiting LPS-induced IκB phosphorylation, IκB degradation, and NF-κB translocation, blocking phosphorylation of ERK1/2 and p38 MAP kinase	-	MAPK/NF-κB	[140]
	-	Primary mouse neurons cell: enhancing cell viability, restoring mitochondrial membrane potential, and reducing ROS production	-	PI3K/Akt	[138]
	-	HSF: decreasing the content of ROS, β-galactosidases, and AGEs and increases mitochondrial membrane potential	-	-	[128]
	-	Human neuroblastoma cells (SH-SY5Y): blocking neuro-inflammation and apoptosis	-	-	[129]
	-	HSF: Combined with vitamin E, enhancing the inhibition of MMP-1 expression after UVA radiation	-	-	[137]
	-	-	60 female subjects: improving skin elasticity, firmness, brightness, tone, and fine lines and wrinkles	-	[132]
	-	-	5 male and 5 female subjects: reduction in markers of inflammatory oxidative damage (e.g., malondialdehyde, protein carbonyls, etc.) and low-density lipoprotein peroxidase protein levels	-	[133]
	-	-	33 healthy male volunteers aged 20 to 30 years old: enhancing skin hydration and elasticity, reducing erythema, melanin, and sebum levels	-	[134]
	-	-	20 volunteers between 40 and 50 years of age: reducing the number of wrinkles and roughness	-	[135]
Astaxanthin	ABTS(IC_50_ 7.7 μg/mL); DPPH(IC_50_ 17.5 μg/mL)	-	-	-	[161]
	ABTS(IC_50_ 17.56 μg/mL); DPPH(IC_50_ 50.93 μg/mL)	-	-	-	[162]
	-	HSF: inhibiting cellular damage caused by free radicals and reducing UVA radiation-induced elevation of IL6 expression	-	-	[151]
	-	Mouse photoreceptor cells (661W): reducing ROS production and attenuating apoptosis	-	PI3K/Akt/Nrf2	[153]
	-	-	Male hairless mice: reducing UV-induced collagen degradation and elastic fiber damage, reducing the expression of MMPs (e.g., MMP-1, MMP-3, MMP-9)	Nrf2/NF-κB/MAPK	[154]
	-	HaCaT keratinocytes: inhibition of cell apoptosis by reducing INOS and COX-2	-	-	[152]
	-	-	Between 30 and 56 years of age, 21 women and 2 men: improving skin elasticity and hydration	-	[149]
	-	-	30 healthy female subjects: improving wrinkles, elasticity, transepidermal water loss, moisture content, and sebum oil levels	-	[150]
Lutein and zeaxanthin	-	Human RPE cell: directly quenching ROS and facilitating glutathione synthesis	-	-	[156]
	-	Rat tracheal epithelial cells: reduction in UVA radiation-induced DNA damage	-	-	[157]
	-	-	Human subjects: improving skin hydration, elasticity, and photoprotective activity	-	[158]
	-	-	Hairless mice: decreasing UVB-induced epidermal hyper-proliferation and acute inflammation in hairless mice	-	[159]
	-	-	Mice: reducing wrinkles and dryness	-	[160]

### 3.2. The Potential of Novel Carotenoids on Anti-Photoaging

#### 3.2.1. C_30_ Carotenoids

C_30_ carotenoids, lipophilic tetraterpenoids composed of six isoprene subunits, are 30-carbon pigments biosynthesized by microorganisms, including *Pseudomonas rhodos* (4,4′-Diapocaroten-4′-al-4-oic acid, C_30_H_36_O_3_), *Staphylococcus aureus* (4,4′-Diapolycopen-4-al (4, 4′-Diapolycopenal), C_30_H_38_O), *Halobacillus halophilus* (Methyl hydroxy-3,4-dehydro-apo-8′-lycopenoate, C_31_H_42_O_3_), *Streptococcus faecium* (4-Hydroxy-4,4′-diaponeurosporene, C_30_H_42_O), and *Heliobacteria spp.* (4,4′-Diaponeurosporene, C_30_H_42_) (Table 2) [163]. Unlike the ubiquitous C_40_ carotenoids, C_30_ variants are phylogenetically restricted, exhibiting structural diversity through hydroxyl, ketone, aldehyde, or carboxyl functional groups. Their radical scavenging efficacy correlates positively with conjugated double bond count (3–13 in known derivatives), wherein extended π-electron delocalization enhances antioxidant potency. Current research prioritizes elucidating their physicochemical properties [163,164,165]. For instance, 4,4′-diaponeurosporene, a C_30_ carotenoid isolated from *Lactiplantibacillus plantarum* subsp. *plantarum* KCCP11226, demonstrated superior DPPH radical scavenging activity compared to BHT [164]. Another study also confirmed that C_30_ carotenoids have good DPPH radical scavenging activity [166].The superior antioxidant capacity of C_30_ carotenoids, combined with their intrinsic lipophilicity that facilitates cutaneous absorption, renders these compounds promising therapeutic agents for counteracting UV-induced photoaging.

#### 3.2.2. C_50_ Carotenoids

C_50_ carotenoids, a novel class of natural pigments predominantly synthesized by halophilic archaea (e.g., *Halorubrum*, *Haloarcula*, and *Haloferax*), mainly existed in hypersaline environments (up to 300 g/L NaCl) [167,168,169]. C_50_ carotenoids include bacterioruberin and its derivatives, such as monoanhydro-bacterioruberin, bisanhydro-bacterioruberin, and trianhydro-bacterioruberin [13,170]. Structurally, bacterioruberin comprises a linear isoprenoid backbone with 13 conjugated double bonds and four hydroxyl groups at both termini. This configuration enables its integration into archaeal cell membranes, where it augments membrane rigidity [171]. In addition, the elongated π-electron conjugation in C_50_ carotenoids confers superior antioxidant capacity compared to C_40_ counterparts (e.g., β-carotene and astaxanthin). Previous studies have shown that C_50_ carotenoids have excellent ABTS and DPPH radical scavenging activity [172,173]. The ABTS radical scavenging assay revealed that bacterioruberin extracts exhibited antioxidant activity 9-fold higher than β-carotene and 26-fold higher than astaxanthin [174]. Furthermore, bacterioruberin demonstrated superior antioxidant efficacy compared to non-pigmented antioxidants, including α-tocopherol, ascorbic acid, and BHT [169]. Beyond its antioxidant properties, bacterioruberin exhibits multiple bioactivities, including antitumor, antihemolytic, antimicrobial, and anti-hepatitis viral [85,86,91]. Mechanistic studies demonstrated that bacterioruberin extracts significantly reduced the viability of HepG2 hepatocellular carcinoma cells and suppressed their metastatic potential [85,86]. Furthermore, bacterioruberin exerts broad-spectrum inhibitory effects against bacterial and fungal pathogens [82,91]. In H_2_O_2_-induced erythrocyte hemolysis models, crude extracts exhibited 3.9- to 6.3-fold higher antihemolytic activity than β-carotene [86]. Notably, bacterioruberin outperformed lamivudine and sofosbuvir, antiviral agents for hepatitis B (HBV) and hepatitis C (HCV) in viral clearance efficacy, as evidenced by superior reduction rates of HBV/HCV titers [87]. Additionally, bacterioruberin extracts were able to protect cells from oxidative DNA damaging agents, protect erythrocytes from H_2_O_2_, and reduce levels of pro-inflammatory cytokines such as TNF-α and IL-6 [86,92,175]. In addition to bacterioruberin, a structurally distinct C_50_ carotenoid, sarcinaxanthin (isolated from *Kocuria palustris*), demonstrated notable in vitro photoprotective efficacy, achieving a sun protection factor (SPF) of 9.36 ± 0.52 [176].These findings underscore that C_50_ carotenoids have broad application potential, particularly in the mitigation of photoaging (Table 3).

## 4. Safety and Bioavailability of Carotenoids

Carotenoids, while offering significant therapeutic potential, present safety and bioavailability challenges that require careful evaluation. This section critically examines their safety profiles and absorption limitations across administration routes.

### 4.1. Oral Administration: Safety Considerations

Excessive oral intake of carotenoid supplements may pose safety risks. One significant concern is carotenemia, a condition characterized by skin yellowing resulting from over-consumption of carotenoids. High-dose carotenoid intake can also induce digestive problems, including bloating, diarrhea, or constipation [177]. Additionally, some studies suggest that high-dose β-carotene supplements might increase the risk of lung cancer in smokers [178]. And the antioxidant properties of carotenoids might potentially mask the symptoms of underlying health issues, such as liver disease. By neutralizing free radicals and reducing oxidative stress, carotenoids may delay the detection of these conditions, thereby postponing diagnosis and treatment.

### 4.2. Topical Application: Efficacy and Safety Profile

**Figure 5:**
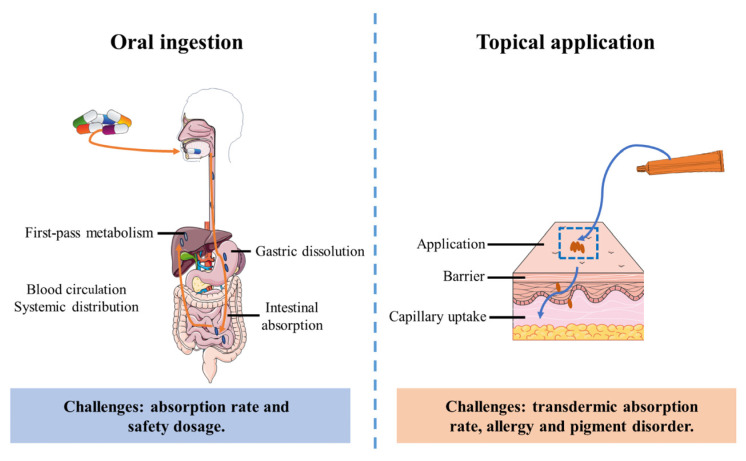
Schematic diagram of carotenoid administration pathways.

Carotenoids can be applied topically, and products containing these pigments are generally regarded as relatively safe. This is because topical application acts directly on the skin surface without involving the digestive system (Figure 5). Nevertheless, there are still some potential safety concerns and side effects associated with topical use. For example, allergic reactions may occur, leading to skin redness, itching, or rashes. Prolonged or excessive use of carotenoid products may also cause changes in skin pigmentation, such as hypopigmentation (loss of color), hyperpigmentation (darkening of the skin), and the formation of discoloration patches. Existing studies have provided some insights into the safety of some carotenoids. For example, Edwards et al. reviewed the genotoxicity and carcinogenicity of astaxanthin in rats by integrating and analyzing data from numerous clinical trials. Their findings indicated that astaxanthin did not exhibit carcinogenicity in the entire life cycle of rat [179]. However, more research is required to fully understand the long-term safety of using carotenoid products, especially at high doses.

Carotenoids encounter several limitations in terms of bioavailability, which can impact their efficacy and practical applications. For oral intake, the primary constraint is their lipid-soluble nature. Carotenoids are insoluble in water and require mixing with lipids for absorption by the body. This property restricts their release from the food matrix and subsequent absorption. During digestion, carotenoids must be emulsified and incorporated into micelles for intestinal absorption. However, this process can be subject to degradation, generally resulting in low bioavailability [180]. Factors such as the presence of dietary fats, the type of food matrix, and individual digestive variations can further affect the absorption efficiency of carotenoids.

Topical delivery confronts distinct barriers, primarily stratum corneum impermeability. Carotenoids need to penetrate the skin barrier for absorption, a process influenced by the skin’s structure and the chemical properties of the carotenoids themselves. The stratum corneum, the outermost layer of the skin, serves as a significant barrier to the penetration of many substances [181]. Additionally, external environmental factors like light, oxygen, and temperature can degrade carotenoids, reducing their stability and effectiveness. The formulation of carotenoid products, including the choice of carrier and excipients, can also impact the release of carotenoids and their ability to penetrate the skin [182]. To address these challenges and enhance the stability and bioavailability of carotenoids, researchers have been exploring various stabilization techniques in recent years. These include microencapsulation [183], liposome preparation [184], and nanoemulsion formulation [185]. These techniques involve physical treatment or chemical modification of carotenoids to enhance their stability within the application system. For example, microencapsulation can protect carotenoids from degradation by encapsulating them in a protective shell, while liposomes and nanoemulsions can improve their solubility and absorption efficiency [186].

## 5. Conclusions and Outlooks

This review systematically reveals the key role of carotenoids in combating skin photoaging induced by UV radiation. Extensive research has demonstrated that C_40_ carotenoids (e.g., β-carotene, lycopene, astaxanthin) can effectively inhibit UV-induced oxidative damage and collagen degradation. These carotenoids achieve protection against UV radiation by scavenging ROS and regulating signaling pathways such as MAPK, Nrf2, and NF-κB. Notably, novel C_50_ carotenoids (especially bacterioruberin) exhibit stronger antioxidant activity than C_40_ carotenoids, allowing potential in anti-photoaging.

Although carotenoids exhibit substantial potential in countering photoaging, numerous challenges and opportunities persist in their application and innovation. Future research should prioritize the following directions: (1) Optimizing delivery systems for enhanced bioavailability. Developing advanced delivery systems is crucial to overcome the bioavailability limitations of carotenoids. Technologies such as microencapsulation, liposome encapsulation, and nanoemulsion formulation can play a pivotal role, which need to be optimized to ensure efficient delivery of carotenoids to their target sites. (2) Unveiling the anti-photoaging mechanisms of novel carotenoids. Novel carotenoids, like bacterioruberin, possess unique anti-photoaging properties that require in-depth exploration. Multi-omics techniques, including genomics, proteomics, and metabolomics, could be employed to comprehensively elucidate their underlying mechanisms. (3) Establishing safety thresholds through dose–response matrices. To ensure the safe use of carotenoids, it is essential to establish accurate safety thresholds. Dose–response matrices should be formulated, considering various factors such as different routes of administration (oral, topical, etc.), individual variability in metabolism, and potential interactions with other substances. (4) Investigating the synergistic effects of carotenoids with other antioxidants. The combination of carotenoids with other antioxidants, such as coenzyme Q10 and vitamin E, may lead to synergistic effects in combating photoaging. These studies will deepen our understanding of the role that carotenoids play in photoaging, thereby providing more robust support for the application of carotenoids in promoting skin health.

## Figures and Tables

**Table 2:** The antioxidant activity of C_30_ carotenoids [166].

Name	DPPH IC_50_ (μM)
4,4′-Diapolycopene	8.7
4,4′-Diaponeurosporene	11.6
4,4′-Diaponeurosporen-4′-al	10.2
4,4′-Diapolycopendial	7.5
4,4′-Diaponeurosporen-4′-oic acid	9.7
4,4′-Diapotorulene	70.3
4,4′-Diapo-β-carotene	77.8

**Table 3:** The anti-photoaging potential of C_50_ carotenoids.

Name	Antioxidant Capacity	In Vitro Experiment	References
Bacterioruberin	ABTS (IC_50_ 9.8 μg/mL), FRAP (IC_50_ 2.1 μg/mL)	-	[172]
	DPPH (IC_50_ 86.67 μg/mL)	-	[173]
	-	Protection of cells against oxidizing DNA damaging agent	[92]
	-	Protection of erythrocytes from H_2_O_2_	[86]
	ABTS (IC_50_ 20.5 μg/mL),	Macrophages: reducing ROS levels and reducing levels of pro-inflammatory cytokines TNF-α and IL-6	[175]
Sarcinaxanthin	^1^O_2_	Displaying an SPF of 9.36 ± 0.52, exhibiting in vitro photoprotective activity	[176]

## References

[1] Millar S.E. (2018). Revitalizing Aging Skin through Diet. Cell.

[2] Zhang S., Duan E. (2018). Fighting against Skin Aging: The Way from Bench to Bedside. Cell Transplant..

[3] Tsatsou F., Trakatelli M., Patsatsi A., Kalokasidis K., Sotiriadis D. (2012). Extrinsic Aging. Dermato-Endocrinology.

[4] Huang A.H., Chien A.L. (2020). Photoaging: A Review of Current Literature. Curr. Dermatol. Rep..

[5] Kammeyer A., Luiten R.M. (2015). Oxidation Events and Skin Aging. Ageing Res. Rev..

[6] Amaro-Ortiz A., Yan B., D’Orazio J. (2014). Ultraviolet Radiation, Aging and the Skin: Prevention of Damage by Topical cAMP Manipulation. Molecules.

[7] Gendrisch F., Esser P.R., Schempp C.M., Wölfle U. (2021). Luteolin as a Modulator of Skin Aging and Inflammation. BioFactors.

[8] Daré R.G., Nakamura C.V., Ximenes V.F., Lautenschlager S.O.S. (2020). Tannic Acid, a Promising Anti-Photoaging Agent: Evidences of Its Antioxidant and Anti-Wrinkle Potentials, and Its Ability to Prevent Photodamage and MMP-1 Expression in L929 Fibroblasts Exposed to UVB. Free. Radic. Biol. Med..

[9] Shin S., Cho S.H., Park D., Jung E. (2020). Anti-skin Aging Properties of Protocatechuic Acid in Vitro and in Vivo. J. Cosmet. Dermatol..

[10] Yutani R., Kikuchi T., Teraoka R., Kitagawa S. (2014). Efficient Delivery and Distribution in Skin of Chlorogenic Acid and Resveratrol Induced by Microemulsion Using Sucrose Laurate. Chem. Pharm. Bull..

[11] Galano A., Álvarez-Diduk R., Ramírez-Silva M.T., Alarcón-Ángeles G., Rojas-Hernández A. (2009). Role of the Reacting Free Radicals on the Antioxidant Mechanism of Curcumin. Chem. Phys..

[12] Ciardullo G., Orlando C., Russo N., Marchese E., Galano A., Marino T., Prejanò M. (2024). On the Dual Role of (+)-Catechin as Primary Antioxidant and Inhibitor of Viral Proteases. Comput. Biol. Med..

[13] Grivard A., Goubet I., Duarte Filho L.M.D.S., Thiéry V., Chevalier S., de Oliveira-Junior R.G., El Aouad N., Guedes da Silva Almeida J.R., Sitarek P., Quintans-Junior L.J. (2022). Archaea Carotenoids: Natural Pigments with Unexplored Innovative Potential. Mar. Drugs.

[14] Dias M.G., Olmedilla-Alonso B., Hornero-Méndez D., Mercadante A.Z., Osorio C., Vargas-Murga L., Meléndez-Martínez A.J. (2018). Comprehensive Database of Carotenoid Contents in Ibero-American Foods. A Valuable Tool in the Context of Functional Foods and the Establishment of Recommended Intakes of Bioactives. J. Agric. Food Chem..

[15] Lu W., Shi Y., Wang R., Su D., Tang M., Liu Y., Li Z. (2021). Antioxidant Activity and Healthy Benefits of Natural Pigments in Fruits: A Review. Int. J. Mol. Sci..

[16] Khan U.M., Sevindik M., Zarrabi A., Nami M., Ozdemir B., Kaplan D.N., Selamoglu Z., Hasan M., Kumar M., Alshehri M.M. (2021). Lycopene: Food Sources, Biological Activities, and Human Health Benefits. Oxidative Med. Cell. Longev..

[17] Rossi N., Grosso C., Delerue-Matos C. (2024). Shrimp Waste Upcycling: Unveiling the Potential of Polysaccharides, Proteins, Carotenoids, and Fatty Acids with Emphasis on Extraction Techniques and Bioactive Properties. Mar. Drugs.

[18] Gallego R., Valdés A., Suárez-Montenegro Z.J., Sánchez-Martínez J.D., Cifuentes A., Ibáñez E., Herrero M. (2022). Anti-Inflammatory and Neuroprotective Evaluation of Diverse Microalgae Extracts Enriched in Carotenoids. Algal Res..

[19] Bolhassani A., Khavari A., Bathaie S.Z. (2014). Saffron and Natural Carotenoids: Biochemical Activities and Anti-Tumor Effects. Biochim. Et Biophys. Acta (Bba)-Rev. Cancer.

[20] Koklesova L., Liskova A., Samec M., Zhai K., Abotaleb M., Ashrafizadeh M., Brockmueller A., Shakibaei M., Biringer K., Bugos O. (2020). Carotenoids in Cancer Metastasis—Status Quo and Outlook. Biomolecules.

[21] Satomi Y. (2017). Antitumor and Cancer-Preventative Function of Fucoxanthin: A Marine Carotenoid. Anticancer. Res..

[22] Hirakida H., Nakamura S., Inagaki S., Tsuji S., Hayashi M., Shimazawa M., Hara H. (2022). Anti-Diabetic Effects of Astaxanthin-Rich Extract Derived from *Paracoccus carotinifaciens* on Pancreatic β Cells. J. Funct. Foods.

[23] Zununi Vahed S., Zuluaga Tamayo M., Rodriguez-Ruiz V., Thibaudeau O., Aboulhassanzadeh S., Abdolalizadeh J., Meddahi-Pellé A., Gueguen V., Barzegari A., Pavon-Djavid G. (2024). Functional Mechanisms of Dietary Crocin Protection in Cardiovascular Models under Oxidative Stress. Pharmaceutics.

[24] Maoka T. (2009). Recent Progress in Structural Studies of Carotenoids in Animals and Plants. Arch. Biochem. Biophys..

[25] Naik A.A., Gadgoli C.H., Naik A.B. (2023). Formulation Containing Phytosomes of Carotenoids from *Nyctanthes Arbor-Tristis* and *Tagetes Patula* Protect D-Galactose Induced Skin Aging in Mice. Clin. Complement. Med. Pharmacol..

[26] Imokawa G. (2019). The Xanthophyll Carotenoid Astaxanthin Has Distinct Biological Effects to Prevent the Photoaging of the Skin Even by Its Postirradiation Treatment. Photochem. Photobiol..

[27] Imokawa G. (2019). Intracellular Signaling Mechanisms Involved in the Biological Effects of the Xanthophyll Carotenoid Astaxanthin to Prevent the Photo-Aging of the Skin in a Reactive Oxygen Species Depletion-Independent Manner: The Key Role of Mitogen and Stress-Activated Protein Kinase 1. Photochem. Photobiol..

[28] Panich U., Sittithumcharee G., Rathviboon N., Jirawatnotai S. (2016). Ultraviolet Radiation-Induced Skin Aging: The Role of DNA Damage and Oxidative Stress in Epidermal Stem Cell Damage Mediated Skin Aging. Stem Cells Int..

[29] Amer R.I., Ezzat S.M., Aborehab N.M., Ragab M.F., Mohamed D., Hashad A., Attia D., Salama M.M., El Bishbishy M.H. (2021). Downregulation of MMP1 Expression Mediates the Anti-Aging Activity of *Citrus sinensis* Peel Extract Nanoformulation in UV Induced Photoaging in Mice. Biomed. Pharmacother..

[30] Gromkowska-Kępka K.J., Puścion-Jakubik A., Markiewicz-Żukowska R., Socha K. (2021). The Impact of Ultraviolet Radiation on Skin Photoaging—Review of in Vitro Studies. J. Cosmet. Dermatol..

[31] Battie C., Jitsukawa S., Bernerd F., Del Bino S., Marionnet C., Verschoore M. (2014). New Insights in Photoaging, UVA Induced Damage and Skin Types. Exp. Dermatol..

[32] Gao S., Guo K., Chen Y., Zhao J., Jing R., Wang L., Li X., Hu Z., Xu N., Li X. (2021). Keratinocyte Growth Factor 2 Ameliorates UVB-Induced Skin Damage via Activating the AhR/Nrf2 Signaling Pathway. Front. Pharmacol..

[33] Karthikeyan R., Kanimozhi G., Prasad N.R., Agilan B., Ganesan M., Srithar G. (2018). Alpha Pinene Modulates UVA-Induced Oxidative Stress, DNA Damage and Apoptosis in Human Skin Epidermal Keratinocytes. Life Sci..

[34] Khan A.Q., Travers J.B., Kemp M.G. (2018). Roles of UVA Radiation and DNA Damage Responses in Melanoma Pathogenesis. Environ. Mol. Mutagen..

[35] Shin J.-W., Kwon S.-H., Choi J.-Y., Na J.-I., Huh C.-H., Choi H.-R., Park K.-C. (2019). Molecular Mechanisms of Dermal Aging and Antiaging Approaches. Int. J. Mol. Sci..

[36] Lai-Cheong J.E., McGrath J.A. (2021). Structure and Function of Skin, Hair and Nails. Medicine.

[37] Mu J., Chen H., Ye M., Zhang X., Ma H. (2022). Acacetin Resists UVA Photoaging by Mediating the SIRT3/ROS/MAPKs Pathway. J. Cell Mol. Med..

[38] Prasedya E.S., Syafitri S.M., Geraldine B.A.F.D., Hamdin C.D., Frediansyah A., Miyake M., Kobayashi D., Hazama A., Sunarpi H. (2019). UVA Photoprotective Activity of Brown Macroalgae Sargassum Cristafolium. Biomedicines.

[39] Lohakul J., Chaiprasongsuk A., Jeayeng S., Saelim M., Muanjumpon P., Thanachaiphiwat S., Tripatara P., Soontrapa K., Lumlerdkij N., Akarasereenont P. (2021). The Protective Effect of Polyherbal Formulation, Harak Formula, on UVA-Induced Photoaging of Human Dermal Fibroblasts and Mouse Skin via Promoting Nrf2-Regulated Antioxidant Defense. Front. Pharmacol..

[40] Galano A., Mazzone G., Alvarez-Diduk R., Marino T., Alvarez-Idaboy J.R., Russo N. (2016). Food Antioxidants: Chemical Insights at the Molecular Level. Annu. Rev. Food Sci. Technol..

[41] Demirci-Çekiç S., Özkan G., Avan A.N., Uzunboy S., Çapanoğlu E., Apak R. (2022). Biomarkers of Oxidative Stress and Antioxidant Defense. J. Pharm. Biomed. Anal..

[42] Catanzaro E., Bishayee A., Fimognari C. (2020). On a Beam of Light: Photoprotective Activities of the Marine Carotenoids Astaxanthin and Fucoxanthin in Suppression of Inflammation and Cancer. Mar. Drugs.

[43] Morales-García B.C., Pérez-González A., Galano A. (2024). Spirochromene and Spiroindene Compounds as Antioxidants. J. Mol. Struct..

[44] Polefka T.G., Meyer T.A., Agin P.P., Bianchini R.J. (2012). Cutaneous Oxidative Stress. J. Cosmet. Dermatol..

[45] Romanhole R.C., Ataide J.A., Moriel P., Mazzola P.G. (2015). Update on Ultraviolet A and B Radiation Generated by the Sun and Artificial Lamps and Their Effects on Skin. Int. J. Cosmet. Sci..

[46] Marionnet C., Pierrard C., Golebiewski C., Bernerd F. (2014). Diversity of Biological Effects Induced by Longwave UVA Rays (UVA1) in Reconstructed Skin. PLoS ONE.

[47] Cadet J., Wagner J.R. (2013). DNA Base Damage by Reactive Oxygen Species, Oxidizing Agents, and UV Radiation. Cold Spring Harb. Perspect. Biol..

[48] Miyata Y., Okada K., Fujimoto A., Hata K.-I., Kagami H., Tomita Y., Ueda M. (2004). The Effect of the Long-Term Cultivation on Telomere Length and Morphology of Cultured Epidermis. J. Dermatol. Sci..

[49] Yadav D.K., Kumar S., Choi E.-H., Chaudhary S., Kim M.-H. (2019). Molecular Dynamic Simulations of Oxidized Skin Lipid Bilayer and Permeability of Reactive Oxygen Species. Sci. Rep..

[50] Ling H., Lou Y. (2005). Total Flavones from Elsholtzia Blanda Reduce Infarct Size during Acute Myocardial Ischemia by Inhibiting Myocardial Apoptosis in Rats. J. Ethnopharmacol..

[51] Andrés Juan C., Pérez de Lastra J.M., Plou Gasca F.J., Pérez-Lebeña E. (2021). The Chemistry of Reactive Oxygen Species (ROS) Revisited: Outlining Their Role in Biological Macromolecules (DNA, Lipids and Proteins) and Induced Pathologies. Int. J. Mol. Sci..

[52] Rosenfeld M.A., Vasilyeva A.D., Yurina L.V., Bychkova A.V. (2018). Oxidation of Proteins: Is It a Programmed Process?. Free Radic. Res..

[53] Grune T. (2018). Redox Regulation in Aging: Role of Protein Aggregates. Free Radic. Biol. Med..

[54] Höhn A., König J., Grune T. (2013). Protein Oxidation in Aging and the Removal of Oxidized Proteins. J. Proteom..

[55] Jang H.-Y., Kim G.-B., Kim J.-M., Kang S.Y., Youn H.-J., Park J., Ro S.Y., Chung E.-Y., Park K.-H., Kim J.-S. (2023). Fisetin Inhibits UVA-Induced Expression of MMP-1 and MMP-3 through the NOX/ROS/MAPK Pathway in Human Dermal Fibroblasts and Human Epidermal Keratinocytes. Int. J. Mol. Sci..

[56] Kciuk M., Gielecińska A., Budzinska A., Mojzych M., Kontek R. (2022). Metastasis and MAPK Pathways. Int. J. Mol. Sci..

[57] Zhang J., Bowden G.T. (2012). Activation of P38 MAP Kinase and JNK Pathways by UVA Irradiation. Photochem. Photobiol. Sci..

[58] Li J., Zhou Y., Wang C. (2007). P38 MAPK in Regulating Cellular Responses to Ultraviolet Radiation. J. Biomed. Sci..

[59] Lasa M., Mahtani K.R., Finch A., Brewer G., Saklatvala J., Clark A.R. (2000). Regulation of Cyclooxygenase 2 mRNA Stability by the Mitogen-Activated Protein Kinase P38 Signaling Cascade. Mol. Cell Biol..

[60] Tanveer M.A., Rashid H., Tasduq S.A. (2023). Molecular Basis of Skin Photoaging and Therapeutic Interventions by Plant-Derived Natural Product Ingredients: A Comprehensive Review. Heliyon.

[61] Yoshioka H., Hino Y., Iwata K., Ogawa T., Yoshioka M., Ishihama N., Adachi H. (2023). Dynamics of Plant Immune MAPK Activity and ROS Signaling in Response to Invaders. Physiol. Mol. Plant Pathol..

[62] Zhang D.D., Lo S.-C., Cross J.V., Templeton D.J., Hannink M. (2004). Keap1 Is a Redox-Regulated Substrate Adaptor Protein for a Cul3-Dependent Ubiquitin Ligase Complex. Mol. Cell Biol..

[63] Wang T., Jian Z., Baskys A., Yang J., Li J., Guo H., Hei Y., Xian P., He Z., Li Z. (2020). MSC-Derived Exosomes Protect against Oxidative Stress-Induced Skin Injury via Adaptive Regulation of the NRF2 Defense System. Biomaterials.

[64] Wu K.C., Cui J.Y., Klaassen C.D. (2011). Beneficial Role of Nrf2 in Regulating NADPH Generation and Consumption. Toxicol. Sci..

[65] Tian F.F., Zhang F.F., Lai X.D., Wang L.J., Yang L., Wang X., Singh G., Zhong J.L. (2011). Nrf2-Mediated Protection against UVA Radiation in Human Skin Keratinocytes. Biosci. Trends.

[66] Hayes J.D., McMahon M., Chowdhry S., Dinkova-Kostova A.T. (2010). Cancer Chemoprevention Mechanisms Mediated through the Keap1-Nrf2 Pathway. Antioxid. Redox Signal.

[67] Saito R., Suzuki T., Hiramoto K., Asami S., Naganuma E., Suda H., Iso T., Yamamoto H., Morita M., Baird L. (2016). Characterizations of Three Major Cysteine Sensors of Keap1 in Stress Response. Mol. Cell Biol..

[68] Hu N., An R., Yu K., Chang Y., Gao W. (2023). PF4 Induces Inflammatory Response through NF-kB Signal Pathway in Rats with Intracerebral Haemorrhage. Folia Neuropathol..

[69] Barber K., Mendonca P., Evans J.A., Soliman K.F.A. (2023). Antioxidant and Anti-Inflammatory Mechanisms of Cardamonin through Nrf2 Activation and NF-kB Suppression in LPS-Activated BV-2 Microglial Cells. Int. J. Mol. Sci..

[70] Pahl H.L. (1999). Activators and Target Genes of Rel/NF-kappaB Transcription Factors. Oncogene.

[71] García-García V.A., Alameda J.P., Page A., Casanova M.L. (2021). Role of NF-κB in Ageing and Age-Related Diseases: Lessons from Genetically Modified Mouse Models. Cells.

[72] Wang Y., Wang L., Wen X., Hao D., Zhang N., He G., Jiang X. (2019). NF-κB Signaling in Skin Aging. Mech. Ageing Dev..

[73] Youssef N.S., Elzaitony A.S., Baky N.A.A. (2022). Diacerein Attenuate LPS-Induced Acute Lung Injury via Inhibiting ER Stress and Apoptosis: Impact on the Crosstalk between SphK1/S1P, TLR4/NFκB/STAT3, and NLRP3/IL-1β Signaling Pathways. Life Sci..

[74] Gong W., Li J., Chen W., Feng F., Deng Y. (2020). Resveratrol Inhibits Lipopolysaccharide-Induced Extracellular Matrix Accumulation and Inflammation in Rat Glomerular Mesangial Cells by SphK1/S1P2/NF-κB Pathway. Diabetes Metab. Syndr. Obes..

[75] Ferraz C.A.A., Grougnet R., Nicolau E., Picot L., de Oliveira Junior R.G. (2022). Carotenoids from Marine Microalgae as Antimelanoma Agents. Mar. Drugs.

[76] Aslanbay Guler B., Saglam-Metiner P., Deniz I., Demirel Z., Yesil-Celiktas O., Imamoglu E. (2023). Aligned with Sustainable Development Goals: Microwave Extraction of Astaxanthin from Wet Algae and Selective Cytotoxic Effect of the Extract on Lung Cancer Cells. Prep. Biochem. Biotechnol..

[77] Li X., Matsumoto T., Takuwa M., Saeed Ebrahim Shaiku Ali M., Hirabashi T., Kondo H., Fujino H. (2020). Protective Effects of Astaxanthin Supplementation against Ultraviolet-Induced Photoaging in Hairless Mice. Biomedicines.

[78] Komatsu T., Sasaki S., Manabe Y., Hirata T., Sugawara T. (2017). Preventive Effect of Dietary Astaxanthin on UVA-Induced Skin Photoaging in Hairless Mice. PLoS ONE.

[79] Arslansoy N., Fidan O., Arslansoy N., Fidan O. (2024). Carotenoids and Their Antioxidant Power. The Power of Antioxidants-Unleashing Nature’s Defense Against Oxidative Stress.

[80] Zeb A., Hussain A. (2020). Chemo-Metric Analysis of Carotenoids, Chlorophylls, and Antioxidant Activity of Trifolium Hybridum. Heliyon.

[81] Chu X., Liu J., Gu W., Tian L., Tang S., Zhang Z., Jiang L., Xu X. (2022). Study of the Properties of Carotenoids and Key Carotenoid Biosynthesis Genes from Deinococcus Xibeiensis R13. Biotechnol. Appl. Biochem..

[82] Sahli K., Gomri M.A., Esclapez J., Gómez-Villegas P., Bonete M.-J., León R., Kharroub K. (2021). Characterization and Biological Activities of Carotenoids Produced by Three Haloarchaeal Strains Isolated from Algerian Salt Lakes. Arch. Microbiol..

[83] Stajčić S., Ćetković G., Čanadanović-Brunet J., Djilas S., Mandić A., Četojević-Simin D. (2015). Tomato Waste: Carotenoids Content, Antioxidant and Cell Growth Activities. Food Chem..

[84] Shimidzu N., Goto M., Miki W. (1996). Carotenoids as Singlet Oxygen Quenchers in Marine Organisms. Fish. Sci..

[85] Abbes M., Baati H., Guermazi S., Messina C., Santulli A., Gharsallah N., Ammar E. (2013). Biological Properties of Carotenoids Extracted from Halobacterium Halobium Isolated from a Tunisian Solar Saltern. BMC Complement. Altern. Med..

[86] Hou J., Cui H.-L. (2018). In Vitro Antioxidant, Antihemolytic, and Anticancer Activity of the Carotenoids from Halophilic Archaea. Curr. Microbiol..

[87] Hegazy G.E., Abu-Serie M.M., Abo-Elela G.M., Ghozlan H., Sabry S.A., Soliman N.A., Abdel-Fattah Y.R. (2020). In Vitro Dual (Anticancer and Antiviral) Activity of the Carotenoids Produced by Haloalkaliphilic Archaeon Natrialba Sp. M6. Sci. Rep..

[88] Shahbazi S., Zargar M., Zolfaghari M.R., Amoozegar M.A. (2023). Carotenoid Pigment of Halophilic Archaeon Haloarcula Sp. A15 Induces Apoptosis of Breast Cancer Cells. Cell Biochem. Funct..

[89] Gómez-Villegas P., Vigara J., Vila M., Varela J., Barreira L., Léon R. (2020). Antioxidant, Antimicrobial, and Bioactive Potential of Two New Haloarchaeal Strains Isolated from Odiel Salterns (Southwest Spain). Biology.

[90] Sheokand P., Tiwari S.K. (2024). Characterization of Carotenoids Extracted from Haloferax Larsenii NCIM 5678 Isolated from Pachpadra Salt Lake, Rajasthan. Extremophiles.

[91] Fariq A., Yasmin A., Jamil M. (2019). Production, Characterization and Antimicrobial Activities of Bio-Pigments by Aquisalibacillus Elongatus MB592, Salinicoccus Sesuvii MB597, and Halomonas Aquamarina MB598 Isolated from Khewra Salt Range, Pakistan. Extremophiles.

[92] Shahmohammadi H.R., Asgarani E., Terato H., Saito T., Ohyama Y., Gekko K., Yamamoto O., Ide H. (1998). Protective Roles of Bacterioruberin and Intracellular KCl in the Resistance of Halobacterium Salinarium against DNA-Damaging Agents. J. Radiat. Res..

[93] Kurniawan R., Taslim N.A., Aman A.M., Syauki A.Y., Bukhari A., Bahar B., Mayulu N., Putra D.E., Syahputra R.A., Kim B. (2025). Pharmacoinformatics and ex vivo Studies of Carotenoids from Green Algae Caulerpa Racemosa as Functional Biomolecules to Modulate Type-2 Diabetes Markers. S. Afr. J. Bot..

[94] Mao M., Lei H., Liu Q., He R., Zuo Z., Zhang N., Zhou C. (2014). Lycopene Inhibits Neointimal Hyperplasia through Regulating Lipid Metabolism and Suppressing Oxidative Stress. Mol. Med. Rep..

[95] Yu L., Gao R., Song X., Li X., Zhu J. (2021). Cardio-Protective and Anti-Atherosclerosis Effect of Crocetin on Vitamin D3 and HFD-Induced Atherosclerosis in Rats. J. Oleo Sci..

[96] Liu S., Mohri S., Tsukamoto M., Yanai Y., Manabe Y., Sugawara T. (2025). Preventive Effects of Dietary Fucoxanthin on Ultraviolet A Induced Photoaging in Hairless Mice. J. Sci. Food Agric..

[97] Shanaida M., Mykhailenko O., Lysiuk R., Hudz N., Balwierz R., Shulhai A., Shapovalova N., Shanaida V., Bjørklund G. (2025). Carotenoids for Antiaging: Nutraceutical, Pharmaceutical, and Cosmeceutical Applications. Pharmaceuticals.

[98] Hata T.R., Scholz T.A., Ermakov I.V., McClane R.W., Khachik F., Gellermann W., Pershing L.K. (2000). Non-Invasive Raman Spectroscopic Detection of Carotenoids in Human Skin. J. Investig. Dermatol..

[99] Meléndez-Martínez A.J. (2019). An Overview of Carotenoids, Apocarotenoids, and Vitamin A in Agro-Food, Nutrition, Health, and Disease. Mol. Nutr. Food Res..

[100] Novikov V.S., Kuzmin V.V., Darvin M.E., Lademann J., Sagitova E.A., Prokhorov K.A., Ustynyuk L.Y., Nikolaeva G.Y. (2022). Relations between the Raman Spectra and Molecular Structure of Selected Carotenoids: DFT Study of α-Carotene, β-Carotene, γ-Carotene and Lycopene. Spectrochim. Acta A Mol. Biomol. Spectrosc..

[101] Lin H.-W., Chang T.-J., Yang D.-J., Chen Y.-C., Wang M., Chang Y.-Y. (2012). Regulation of Virus-Induced Inflammatory Response by β-Carotene in RAW264.7 Cells. Food Chem..

[102] Dembińska-Kieć A., Malczewska-Malec M., Polus A., Kieć-Wilk B., Grzybowska J., Stachura J., Dyduch G., Pryjma J., Skrzeczyńska J., Langman T. (2006). β-Carotene and Angiogenesis. Pure Appl. Chem..

[103] Białek-Bylka G.E., Jazurek B., Dedic R., Hala J., Skrzypczak A. (2003). Unique Spectroscopic Properties of Synthetic 15-Cis Beta-Carotene, an Important Compound in Photosynthesis, and a Medicine for Photoprotective Function. Cell Mol. Biol. Lett..

[104] Cui B., Liu S., Wang Q., Lin X. (2012). Effect of β-Carotene on Immunity Function and Tumour Growth in Hepatocellular Carcinoma Rats. Molecules.

[105] Cho S., Lee D.H., Won C.-H., Kim S.M., Lee S., Lee M.-J., Chung J.H. (2010). Differential Effects of Low-Dose and High-Dose Beta-Carotene Supplementation on the Signs of Photoaging and Type I Procollagen Gene Expression in Human Skin in Vivo. Dermatology.

[106] Lee J., Jiang S., Levine N., Watson R.R. (2000). Carotenoid Supplementation Reduces Erythema in Human Skin after Simulated Solar Radiation Exposure. Proc. Soc. Exp. Biol. Med..

[107] Minami Y., Kawabata K., Kubo Y., Arase S., Hirasaka K., Nikawa T., Bando N., Kawai Y., Terao J. (2009). Peroxidized Cholesterol-Induced Matrix Metalloproteinase-9 Activation and Its Suppression by Dietary Beta-Carotene in Photoaging of Hairless Mouse Skin. J. Nutr. Biochem..

[108] Schagen S.K., Zampeli V.A., Makrantonaki E., Zouboulis C.C. (2012). Discovering the Link between Nutrition and Skin Aging. Dermato-Endocrinology.

[109] White A.L., Jahnke L.S. (2002). Contrasting Effects of UV-A and UV-B on Photosynthesis and Photoprotection of Beta-Carotene in Two *Dunaliella* spp.. Plant Cell Physiol..

[110] Balić A., Mokos M. (2019). Do We Utilize Our Knowledge of the Skin Protective Effects of Carotenoids Enough?. Antioxidants.

[111] Camera E., Mastrofrancesco A., Fabbri C., Daubrawa F., Picardo M., Sies H., Stahl W. (2009). Astaxanthin, Canthaxanthin and Beta-Carotene Differently Affect UVA-Induced Oxidative Damage and Expression of Oxidative Stress-Responsive Enzymes. Exp. Dermatol..

[112] Wertz K., Seifert N., Hunziker P.B., Riss G., Wyss A., Hunziker W., Goralczyk R. (2006). β-Carotene Interference with UVA-Induced Gene Expression by Multiple Pathways. Pure Appl. Chem..

[113] Ben-Dor A., Steiner M., Gheber L., Danilenko M., Dubi N., Linnewiel K., Zick A., Sharoni Y., Levy J. (2005). Carotenoids Activate the Antioxidant Response Element Transcription System. Mol. Cancer Ther..

[114] Xu G., Ma T., Zhou C., Zhao F., Peng K., Li B. (2022). β-Carotene Attenuates Apoptosis and Autophagy via PI3K/AKT/mTOR Signaling Pathway in Necrotizing Enterocolitis Model Cells IEC-6. Evidence-Based Complement. Altern. Med..

[115] Wu T., Xie Y., Wu Z., Li Y., Jiang M., Yu H., Li X., Wang J., Zhou E., Yang Z. (2023). β-Carotene Protects Mice against Lipopolysaccharide and D-Galactosamine Induced Acute Liver Injury via Regulation of NF-κB, MAPK, and Nrf2 Signaling. J. Oleo Sci..

[116] Zheng W.V., Xu W., Li Y., Qin J., Zhou T., Li D., Xu Y., Cheng X., Xiong Y., Chen Z. (2022). Anti-Aging Effect of β-Carotene through Regulating the KAT7-P15 Signaling Axis, Inflammation and Oxidative Stress Process. Cell Mol. Biol. Lett..

[117] Wertz K., Hunziker P.B., Seifert N., Riss G., Neeb M., Steiner G., Hunziker W., Goralczyk R. (2005). Beta-Carotene Interferes with Ultraviolet Light A-Induced Gene Expression by Multiple Pathways. J. Investig. Dermatol..

[118] Wertz K., Seifert N., Hunziker P.B., Riss G., Wyss A., Lankin C., Goralczyk R. (2004). β-Carotene Inhibits UVA-Induced Matrix Metalloprotease 1 and 10 Expression in Keratinocytes by a Singlet Oxygen-Dependent Mechanism. Free Radic. Biol. Med..

[119] Bayerl C. (2008). Beta-Carotene in Dermatology: Does It Help?. Acta Dermatoven APA.

[120] Grabowska M., Wawrzyniak D., Rolle K., Chomczyński P., Oziewicz S., Jurga S., Barciszewski J. (2019). Let Food Be Your Medicine: Nutraceutical Properties of Lycopene. Food Funct..

[121] Rao A.V., Rao L.G. (2007). Carotenoids and Human Health. Pharmacol. Res..

[122] Wang Z., Sun J., Yang Q., Yang J. (2020). Metabolic Engineering Escherichia Coli for the Production of Lycopene. Molecules.

[123] Arathi B.P., Sowmya P.R.-R., Vijay K., Baskaran V., Lakshminarayana R. (2015). Metabolomics of Carotenoids: The Challenges and Prospects—A Review. Trends Food Sci. Technol..

[124] Kim Y.-S., Lee J.-H., Kim N.-H., Yeom S.-J., Kim S.-W., Oh D.-K. (2011). Increase of Lycopene Production by Supplementing Auxiliary Carbon Sources in Metabolically Engineered Escherichia Coli. Appl. Microbiol. Biotechnol..

[125] Kang C.K., Jeong S.-W., Yang J.E., Choi Y.J. (2020). High-Yield Production of Lycopene from Corn Steep Liquor and Glycerol Using the Metabolically Engineered Deinococcus Radiodurans R1 Strain. J. Agric. Food Chem..

[126] Di Mascio P., Kaiser S., Sies H. (1989). Lycopene as the Most Efficient Biological Carotenoid Singlet Oxygen Quencher. Arch. Biochem. Biophys..

[127] Liu C.-C., Huang C.-C., Lin W.-T., Hsieh C.-C., Huang S.-Y., Lin S.-J., Yang S.-C. (2005). Lycopene Supplementation Attenuated Xanthine Oxidase and Myeloperoxidase Activities in Skeletal Muscle Tissues of Rats after Exhaustive Exercise. Br. J. Nutr..

[128] Li J., Zeng X., Yang X., Ding H. (2022). Lycopene Ameliorates Skin Aging by Regulating the Insulin Resistance Pathway and Activating SIRT1. Food Funct..

[129] Almeer R., Alyami N.M. (2025). Effect of Lycopene on TiO_2_ Nanoforms Induced Oxidative Stress and Neuroinflammation in SH-SY5Y Cells: An in Vitro Study. Drug Chem. Toxicol..

[130] Honda M. (2023). Z-Isomers of Lycopene and β-Carotene Exhibit Greater Skin-Quality Improving Action than Their All-E-Isomers. Food Chem..

[131] Stahl W., Heinrich U., Aust O., Tronnier H., Sies H. (2006). Lycopene-Rich Products and Dietary Photoprotection. Photochem. Photobiol. Sci..

[132] Tarshish E., Hermoni K., Sharoni Y., Muizzuddin N. (2022). Effect of Lumenato Oral Supplementation on Plasma Carotenoid Levels and Improvement of Visual and Experiential Skin Attributes. J. Cosmet. Dermatol..

[133] Chernyshova M.P., Pristenskiy D.V., Lozbiakova M.V., Chalyk N.E., Bandaletova T.Y., Petyaev I.M. (2019). Systemic and Skin-Targeting Beneficial Effects of Lycopene-Enriched Ice Cream: A Pilot Study. J. Dairy. Sci..

[134] Sohail M., Baig M.M.F.A., Akhtar N., Chen Y., Xie B., Li B. (2022). Topical Lycopene Emulgel Significantly Improves Biophysical Parameters of Human Skin. Eur. J. Pharm. Biopharm..

[135] Darvin M., Patzelt A., Gehse S., Schanzer S., Benderoth C., Sterry W., Lademann J. (2008). Cutaneous Concentration of Lycopene Correlates Significantly with the Roughness of the Skin. Eur. J. Pharm. Biopharm..

[136] Rizwan M., Rodriguez-Blanco I., Harbottle A., Birch-Machin M.A., Watson R.E.B., Rhodes L.E. (2011). Tomato Paste Rich in Lycopene Protects against Cutaneous Photodamage in Humans in Vivo: A Randomized Controlled Trial. Br. J. Dermatol..

[137] Offord E.A., Gautier J.-C., Avanti O., Scaletta C., Runge F., Krämer K., Applegate L.A. (2002). Photoprotective Potential of Lycopene, β-Carotene, Vitamin E, Vitamin C and Carnosic Acid in UVA-Irradiated Human Skin Fibroblasts. Free Radic. Biol. Med..

[138] Huang C., Wen C., Yang M., Gan D., Fan C., Li A., Li Q., Zhao J., Zhu L., Lu D. (2019). Lycopene Protects against T-BHP-Induced Neuronal Oxidative Damage and Apoptosis via Activation of the PI3K/Akt Pathway. Mol. Biol. Rep..

[139] Fang Y., Ou S., Wu T., Zhou L., Tang H., Jiang M., Xu J., Guo K. (2020). Lycopene Alleviates Oxidative Stress via the PI3K/Akt/Nrf2pathway in a Cell Model of Alzheimer’s Disease. PeerJ.

[140] Feng D., Ling W.-H., Duan R.-D. (2010). Lycopene Suppresses LPS-Induced NO and IL-6 Production by Inhibiting the Activation of ERK, p38MAPK, and NF-κB in Macrophages. Inflamm. Res..

[141] Guerin M., Huntley M.E., Olaizola M. (2003). *Haematococcus* Astaxanthin: Applications for Human Health and Nutrition. Trends Biotechnol..

[142] Yuan J.P., Peng J., Yin K., Wang J.H. (2011). Potential Health-Promoting Effects of Astaxanthin: A High-Value Carotenoid Mostly from Microalgae. Mol. Nutr. Food Res..

[143] Mutale-Joan C., El Arroussi H. (2025). Biotechnological Strategies Overcoming Limitations to H. Pluvialis-Derived Astaxanthin Production and Morocco’s Potential. Crit. Rev. Food Sci. Nutr..

[144] Cunha F.F.M.D., Tonon A.P., Machado F., Travassos L.R., Grazzia N., Possatto J.F., Sant’ana A.K.C.D., Lopes R.D.M., Rodrigues T., Miguel D.C. (2024). Astaxanthin Induces Autophagy and Apoptosis in Murine Melanoma B16F10-Nex2 Cells and Exhibits Antitumor Activity in Vivo. J. Chemother..

[145] Shanmugapriya K., Kim H., Kang H.W. (2019). In Vitro Antitumor Potential of Astaxanthin Nanoemulsion against Cancer Cells via Mitochondrial Mediated Apoptosis. Int. J. Pharm..

[146] Gowd V., Xiao J., Wang M., Chen F., Cheng K.-W. (2021). Multi-Mechanistic Antidiabetic Potential of Astaxanthin: An Update on Preclinical and Clinical Evidence. Mol. Nutr. Food Res..

[147] Kishimoto Y., Yoshida H., Kondo K. (2016). Potential Anti-Atherosclerotic Properties of Astaxanthin. Mar. Drugs.

[148] Kohandel Z., Farkhondeh T., Aschner M., Pourbagher-Shahri A.M., Samarghandian S. (2022). Anti-Inflammatory Action of Astaxanthin and Its Use in the Treatment of Various Diseases. Biomed. Pharmacother..

[149] Ito N., Seki S., Ueda F. (2018). The Protective Role of Astaxanthin for UV-Induced Skin Deterioration in Healthy People—A Randomized, Double-Blind, Placebo-Controlled Trial. Nutrients.

[150] Tominaga K., Hongo N., Karato M., Yamashita E. (2012). Cosmetic Benefits of Astaxanthin on Humans Subjects. Acta Biochim. Pol..

[151] Suganuma K., Nakajima H., Ohtsuki M., Imokawa G. (2010). Astaxanthin Attenuates the UVA-Induced up-Regulation of Matrix-Metalloproteinase-1 and Skin Fibroblast Elastase in Human Dermal Fibroblasts. J. Dermatol. Sci..

[152] Yoshihisa Y., Rehman M.U., Shimizu T. (2014). Astaxanthin, a Xanthophyll Carotenoid, Inhibits Ultraviolet-Induced Apoptosis in Keratinocytes. Exp. Dermatol..

[153] Lai T.-T., Yang C.-M., Yang C.-H. (2020). Astaxanthin Protects Retinal Photoreceptor Cells against High Glucose-Induced Oxidative Stress by Induction of Antioxidant Enzymes via the PI3K/Akt/Nrf2 Pathway. Antioxidants.

[154] Hama S., Takahashi K., Inai Y., Shiota K., Sakamoto R., Yamada A., Tsuchiya H., Kanamura K., Yamashita E., Kogure K. (2012). Protective Effects of Topical Application of a Poorly Soluble Antioxidant Astaxanthin Liposomal Formulation on Ultraviolet-Induced Skin Damage. J. Pharm. Sci..

[155] Li R., Turner S.D., Brautigan D.L. (2015). Xanthophylls Lutein and Zeaxanthin Modify Gene Expression and Induce Synthesis of Hyaluronan in Keratinocyte Model of Human Skin. Biochem. Biophys. Rep..

[156] Murillo A.G., Hu S., Fernandez M.L. (2019). Zeaxanthin: Metabolism, Properties, and Antioxidant Protection of Eyes, Heart, Liver, and Skin. Antioxidants.

[157] Santocono M., Zurria M., Berrettini M., Fedeli D., Falcioni G. (2006). Influence of Astaxanthin, Zeaxanthin and Lutein on DNA Damage and Repair in UVA-Irradiated Cells. J. Photochem. Photobiol. B Biol..

[158] Palombo P., Fabrizi G., Ruocco V., Ruocco E., Fluhr J., Roberts R., Morganti P. (2007). Beneficial Long-Term Effects of Combined Oral/Topical Antioxidant Treatment with the Carotenoids Lutein and Zeaxanthin on Human Skin: A Double-Blind, Placebo-Controlled Study. Skin Pharmacol. Physiol..

[159] González S., Astner S., An W., Pathak M.A., Goukassian D. (2003). Dietary Lutein/Zeaxanthin Decreases Ultraviolet B-Induced Epidermal Hyperproliferation and Acute Inflammation in Hairless Mice. J. Investig. Dermatol..

[160] Roberts R.L., Green J., Lewis B. (2009). Lutein and Zeaxanthin in Eye and Skin Health. Clin. Dermatol..

[161] Chintong S., Phatvej W., Rerk-Am U., Waiprib Y., Klaypradit W. (2019). In Vitro Antioxidant, Antityrosinase, and Cytotoxic Activities of Astaxanthin from Shrimp Waste. Antioxidants.

[162] Abd El-Ghany M.N., Hamdi S.A., Elbaz R.M., Aloufi A.S., El Sayed R.R., Ghonaim G.M., Farahat M.G. (2023). Development of a Microbial-Assisted Process for Enhanced Astaxanthin Recovery from Crab Exoskeleton Waste. Fermentation.

[163] Siziya I.N., Hwang C.Y., Seo M.-J. (2022). Antioxidant Potential and Capacity of Microorganism-Sourced C_30_ Carotenoids—A Review. Antioxidants.

[164] Kim M., Seo D.-H., Park Y.-S., Cha I.-T., Seo M.-J. (2019). Isolation of Lactobacillus Plantarum Subsp. Plantarum Producing C_30_ Carotenoid 4,4’-Diaponeurosporene and the Assessment of Its Antioxidant Activity. J. Microbiol. Biotechnol..

[165] Hwang C.Y., Cho E.-S., Yoon D.J., Seo M.-J. (2023). Probiotic and Antioxidant Properties of C_30_ Carotenoid-Producing Lactiplantibacillus Plantarum Isolated from Kimchi. Food Sci. Biotechnol..

[166] Kim S.H., Kim M.S., Lee B.Y., Lee P.C. (2016). Generation of Structurally Novel Short Carotenoids and Study of Their Biological Activity. Sci. Rep..

[167] Naziri D., Hamidi M., Hassanzadeh S., Tarhriz V., Zanjani B.M., Nazemyieh H., Hejazi M.A., Hejazi M.S. (2014). Analysis of Carotenoid Production by Halorubrum Sp. TBZ126; an Extremely Halophilic Archeon from Urmia Lake. Adv. Pharm. Bull..

[168] Alvares J.J., Furtado I.J. (2021). Characterization of Multicomponent Antioxidants from Haloferax Alexandrinus GUSF-1 (KF796625). 3 Biotech.

[169] Squillaci G., Parrella R., Carbone V., Minasi P., La Cara F., Morana A. (2017). Carotenoids from the Extreme Halophilic Archaeon Haloterrigena Turkmenica: Identification and Antioxidant Activity. Extremophiles.

[170] Moopantakath J., Imchen M., Anju V.T., Busi S., Dyavaiah M., Martínez-Espinosa R.M., Kumavath R. (2023). Bioactive Molecules from Haloarchaea: Scope and Prospects for Industrial and Therapeutic Applications. Front. Microbiol..

[171] Flegler A., Lipski A. (2021). The C50 Carotenoid Bacterioruberin Regulates Membrane Fluidity in Pink-Pigmented Arthrobacter Species. Arch. Microbiol..

[172] Hwang C.Y., Cho E.-S., Kim S., Kim K., Seo M.-J. (2024). Optimization of Bacterioruberin Production from Halorubrum Ruber and Assessment of Its Antioxidant Potential. Microb. Cell Factories.

[173] Bouhamed S.B.H., Chaari M., Baati H., Zouari S., Ammar E. (2024). Extreme Halophilic Archaea: Halobacterium Salinarum Carotenoids Characterization and Antioxidant Properties. Heliyon.

[174] Flores N., Hoyos S., Venegas M., Galetović A., Zúñiga L.M., Fábrega F., Paredes B., Salazar-Ardiles C., Vilo C., Ascaso C. (2020). *Haloterrigena* Sp. Strain SGH1, a Bacterioruberin-Rich, Perchlorate-Tolerant Halophilic Archaeon Isolated From Halite Microbial Communities, Atacama Desert, Chile. Front. Microbiol..

[175] Ávila-Román J., Gómez-Villegas P., de Carvalho C.C.C.R., Vigara J., Motilva V., León R., Talero E. (2023). Up-Regulation of the Nrf2/HO-1 Antioxidant Pathway in Macrophages by an Extract from a New Halophilic Archaea Isolated in Odiel Saltworks. Antioxidants.

[176] Mendes-Silva T.D.C.D., Vidal E.E., de Souza R.D.F.R., da Cunha Schmidt K., Mendes P.V.D., da Silva Andrade R.F., da Silva Oliveira F.G., de Lucena B.T.L., de Oliveira M.B.M., dos Santos Correia M.T. (2021). Production of Carotenoid Sarcinaxanthin by Kocuria Palustris Isolated from Northeastern Brazil Caatinga Soil and Their Antioxidant and Photoprotective Activities. Electron. J. Biotechnol..

[177] Wang J., Kong W., Liu M., Wang Y., Zheng Y., Zhou Y. (2023). Association between Dietary Carotenoids Intake and Chronic Constipation in American Men and Women Adults: A Cross-Sectional Study. BMC Public Health.

[178] Bates C.A., Vincent M.J., Buerger A.N., Santamaria A.B., Maier A., Jack M. (2024). Investigating the Relationship between β-Carotene Intake from Diet and Supplements, Smoking, and Lung Cancer Risk. Food Chem. Toxicol..

[179] Edwards J.A., Bellion P., Beilstein P., Rümbeli R., Schierle J. (2016). Review of Genotoxicity and Rat Carcinogenicity Investigations with Astaxanthin. Regul. Toxicol. Pharmacol..

[180] Ramos-Souza C., De Rosso V.V., Campos Chisté R., Helena de Aguiar Andrade E., Santana de Oliveira M. (2024). Recent Approaches for the Bioaccessibility and Bioavailability of Carotenoids. Carotenoids: Trends and Advances.

[181] Bouwstra J.A., Ponec M. (2006). The Skin Barrier in Healthy and Diseased State. Biochim. Biophys. Acta (BBA)-Biomembr..

[182] Liu W.-Y., Hsieh Y.-S., Ko H.-H., Wu Y.-T. (2023). Formulation Approaches to Crystalline Status Modification for Carotenoids: Impacts on Dissolution, Stability, Bioavailability, and Bioactivities. Pharmaceutics.

[183] de Freitas Santos P.D., Rubio F.T.V., da Silva M.P., Pinho L.S., Favaro-Trindade C.S. (2021). Microencapsulation of Carotenoid-Rich Materials: A Review. Food Res. Int..

[184] Tan C., Xue J., Lou X., Abbas S., Guan Y., Feng B., Zhang X., Xia S. (2014). Liposomes as Delivery Systems for Carotenoids: Comparative Studies of Loading Ability, Storage Stability and in Vitro Release. Food Funct..

[185] Jalali-Jivan M., Rostamabadi H., Assadpour E., Tomas M., Capanoglu E., Alizadeh-Sani M., Kharazmi M.S., Jafari S.M. (2022). Recent Progresses in the Delivery of β-Carotene: From Nano/Microencapsulation to Bioaccessibility. Adv. Colloid. Interface Sci..

[186] Luo X., Zhou Y., Bai L., Liu F., Deng Y., McClements D.J. (2017). Fabrication of β-Carotene Nanoemulsion-Based Delivery Systems Using Dual-Channel Microfluidization: Physical and Chemical Stability. J. Colloid. Interface Sci..

